# ADAR1 Is Essential for Smooth Muscle Homeostasis and Vascular Integrity

**DOI:** 10.3390/cells13151257

**Published:** 2024-07-26

**Authors:** Dunpeng Cai, Shi-You Chen

**Affiliations:** 1 Departments of Surgery, University of Missouri School of Medicine, Columbia, MO 65212, USA; dccfn@mail.missouri.edu; 2 The Research Service, Harry S. Truman Memorial Veterans Hospital, Columbia, MO 65201, USA

**Keywords:** vascular smooth muscle, apoptosis, adenosine deaminases acting on RNA 1, elastin, fibrillin

## Abstract

Vascular smooth muscle cells (VSMCs) play a critical role in maintaining vascular integrity. VSMC dysfunction leads to numerous vascular diseases. Adenosine deaminases acting on RNA 1 (ADAR1), an RNA editing enzyme, has shown both RNA editing and non-editing functions. Global deletion of ADAR1 causes embryonic lethality, but the phenotype of homozygous ADAR1 deletion specifically in SMCs (ADAR1sm-/-) remains to be determined. By crossing ADAR1fl/fl mice with Myh11-CreERT2 mice followed by Tamoxifen induction, we found that ADAR1sm-/- leads to lethality in adult mice 14 days after the induction. Gross examination revealed extensive hemorrhage and detrimental vascular damage in different organs. Histological analyses revealed destruction of artery structural integrity with detachment of elastin laminae from VSMCs in ADAR1sm-/- aortas. Furthermore, ADAR1sm-/- resulted in severe VSMC apoptosis and mitochondrial dysfunction. RNA sequencing analyses of ADAR1sm-/- aorta segments demonstrated profound transcriptional alteration of genes impacting vascular health including a decrease in fibrillin-1 expression. More importantly, ADAR1sm-/- disrupts the elastin and fibrillin-1 interaction, a molecular event essential for artery structure. Our results indicate that ADAR1 plays a critical role in maintaining SMC survival and vascular stability and resilience.

## 1. Introduction

Vascular smooth muscle cells (VSMCs) play important roles in the physiological functioning of blood vessels. In healthy blood vessels of an adult organism, VSMCs ensure that the blood vessels contract and relax and make a marked contribution to the regulation of blood circulation [1]. In addition, VSMCs are responsible for producing all the important extracellular matrix (ECM) proteins including elastin, fibrillin, collagens, and many other ECM proteins that are critically important for maintaining artery structural integrity and functions. VSMC degeneration or dysfunction contributes to the pathological vascular remodeling involved in a large number of cardiovascular diseases such as thoracic and abdominal aortic aneurysms including Marfan syndrome, atherosclerosis, hypertension, vein graft stenosis, restenosis after angioplasty, and cardiac allograft vasculopathy. However, the molecular mechanisms underlying VSMC survival and its interactions with ECM remain largely unknown [2,3,4,5,6,7,8].

Adenosine deaminases acting on RNA 1 (ADAR1) is an RNA editing enzyme that plays pivotal roles in the RNA editing process by converting adenosine (A) to inosine (I) in double-stranded RNA substrates [9,10,11,12,13]. Beyond its role in A-to-I editing, ADAR1 has also shown non-editing functions by interacting with other proteins such as PKR and DICER [14,15,16,17,18]. ADAR1 has been implicated in various pathologies, including type I interferonopathies, where it modulates immune responses. It also influences cancer progression by affecting cell proliferation and invasion [9,13,19] and plays important roles in the development of cardiovascular diseases through its RNA editing and editing-independent functions [20,21,22,23,24,25].

Global knockout of ADAR1 in mice results in embryonic lethality between E11.5 and E12.5 because of severe liver defects [26,27,28]. However, heterozygous ADAR1 deficiency in mice does not cause apparent defects but attenuates the phenotypic modulation of vascular smooth muscle cells (VSMCs) during vascular injury-caused neointima formation and aneurysm development [22,29]. Surprisingly, homozygous ADAR1 deletion in adult SMCs (ADAR1sm-/-) causes lethality in mice. In this study, we analyzed the phenotype of ADAR1sm-/- mice and explored the mechanisms underlying the causes of lethality. We found that ADAR1sm-/- causes extensive hemorrhage and detrimental vascular damage in different organs. Further analyses showed that large artery structural integrity is damaged, as shown by the detachment of VSMCs from elastin laminae in ADAR1sm-/- aortas. Additionally, severe VSMC apoptosis and mitochondrial dysfunction were found in ADAR1sm-/- aortas. RNA sequencing analyses of ADAR1sm-/- aorta segments showed profound transcriptional alterations for genes impacting vascular health including decreased fibrillin-1 expression. More importantly, ADAR1sm-/- disrupted the elastin and fibrillin-1 interaction, a molecular event essential for artery structure. Our results indicate that ADAR1 plays a critical role in maintaining SMC survival and vascular stability and resilience.

## 2. Materials and Methods

### 2.1. Mice

Male ADAR1fl/fl mice (B6.129-Adartm1Knk/Mmjax) were purchased from the Jackson Laboratory (MMRRC Strain # 034619-JAX). Myh11-CreERT2 mice were obtained from Dr. Gary K Owens [30]. The ADAR1fl/fl mice were cross-bred with Myh-CreERT2 mice to generate ADAR1 SMC-specific knockout (ADAR1sm-/-) after tamoxifen induction (1 mg/day, i.p. for 5 days) [30]. Only male mice were used in this study because the Cre gene is located on the Y chromosome. All mice were in C57BL6 genetic background. The animals were housed under conventional conditions in animal care facilities and received humane care in compliance with the Principles of Laboratory Animal Care formulated by the National Society for Medical Research and the Guide for the Care and Use of Laboratory Animals. All animal procedures were approved by the Institutional Animal Care and Use Committee of the University of Missouri. Animals were randomly grouped, and the operators were blinded to the grouping. The number of animals used was determined by power calculation based on prior experience.

### 2.2. Histopathology Staining

Aorta tissues were fixed in 4% paraformaldehyde in PBS (PFA, AAJ61899AK, ThermoFisher, Waltham, MA, USA) and embedded in paraffin (8330K, Histoplast Paraffin, Epredia™, Portsmouth, NH, USA) or Optimal cutting temperature compound (OCT, 23-730-571, Fisher, Waltham, MA, USA). Tissue sections (5 µm thick) were stained with hematoxylin–eosin (H&E) or Verhoeff’s elastic staining (EVG) for histopathological analyses. H&E and EVG staining were performed using commercial kits (SKU#KTHNEPT, StatLab for H&E kit, McKinney, TX, USA; and HT25A, Sigma for EVG kit, St. Louis, MO, USA) according to the manufacturers’ protocols. Images were acquired with a Keyence fluorescence microscope. The images with average staining signals were selected as representative pictures and used in the figure panels.

### 2.3. Western Blotting

Thoracic aorta tissues were lysed in RIPA lysis buffer (1% Nonidet P-40, 0.1% sodium dodecyl sulfate (SDS), 0.5% sodium deoxycholate, 1 mM sodium orthovanadate, and protease inhibitors) to extract total proteins. To detect cytochrome C, tissue homogenates in RIPA were mixed with an equal volume of sucrose solution (2 mol/L, Sigma, 57-50-1, St. Louis, MO, USA) in a 1.5 mL tube. The mixture was then centrifugated at 7000× *g* for 10 min at 4 °C to separate the mitochondria from the supernatant. The supernatant was collected for further analysis [31,32]. Samples were separated on SDS-polyacrylamide gels and electro-transferred onto nitrocellulose membranes (45-004-001, Amersham Biosciences, Little Chalfont, Buckinghamshire, UK). After blocking with 5% BSA, the membranes were incubated with various primary antibodies at 4 °C overnight. The primary antibodies that were used included Cleaved Caspase 3 (Cell Signaling, #9661, Danvers, MA, USA), Cleaved PARP (#5625, Cell Signaling, Danvers, MA, USA), Bcl-1 (#2764, Cell Signaling, Danvers, MA, USA), Bax (#2772, Cell Signaling, Danvers, MA, USA), cytochrome c (sc-13156, Santa Cruz, Dallas, TX, USA), and GAPDH (60004-1-Ig, Proteintech, Rosemont, IL, USA). The membranes were then incubated with IRDye secondary antibodies (926-32212 and 926-32213, LI-COR Biosciences, Lincoln, NE, USA) at room temperature for 1 h. The protein expression was detected by the Odyssey CLx Imaging System (LICOR Biosciences, Lincoln, NE, USA). Original images can be found in Supplementary Materials.

### 2.4. Co-Immunoprecipitation (Co-IP)

Protein A/G-agarose beads (Santa Cruz, CA) were incubated with normal IgG, Elastin (sc-166543, Santa Cruz), or Fibrillin-1 (29425-1-AP, ProteinTech, Rosemont, IL, USA) antibody at 4 °C for 2 h. Thoracic aorta tissues were homogenized and lysed in 500 µL Co-IP lysis buffer (26149, Pierce, Rockford, IL, USA) on ice for 5 min, and the supernatants were incubated with antibody-conjugated beads at 4 °C overnight. After washing with the Co-IP buffer, proteins were eluted from the beads and boiled in the SDS loading buffer. Western blotting was performed to detect the presence of relevant proteins.

### 2.5. Proximity Ligation Assay (PLA)

PLA was performed by using the reagents provided in the Duo-link PLA kit (DUO92101-1KT, Sigma-Aldrich, St. Louis, MO, USA) according to the manufacturer’s instructions with minor modifications. Briefly, mouse control aorta or AAA tissue sections (5 µm) were deparaffinized, re-hydrated, permeabilized with Triton 0.3% (in PBS), and then incubated with blocking solution for 45 min followed by incubation with rabbit anti-elastin (ab21610, Abcam, Cambridge, UK) and mouse anti-fibrillin-1 (MA5-12770, Invitrogen, Carlsbad, CA, USA) antibodies at 4 °C overnight. After washing with Buffer A three times, the sections were incubated with secondary antibodies conjugated with PLA DNA probes at 37 °C for 1 h. Following 4 × 10 min washings and a rinse at 37 °C with Buffer A, sections were incubated with ligation buffer containing oligonucleotides that can hybridize to PLA probes to form a rolling circle DNA strand by DNA ligase, which was incubated at 37 °C for 30 min. Subsequently, the sections were washed with Buffer A at 37 °C and incubated with the amplification-detection solution containing DNA polymerase for rolling circle amplification at 37 °C for 100 min. Then, the sections were washed with Buffer B four times followed by washing four times with 0.01× Buffer B. Finally, the sections were mounted with mounting buffer containing DAPI under coverslips and observed with a fluorescence microscope (Keyence Corporation of America, Itasca, IL, USA). The PLA spots were counted with Image J (1.54 h), and the mean spot number/cell was calculated for each sample. Rabbit and mouse IgG antibodies were used as negative controls.

### 2.6. Flowcytometry and Tissue Digestion

A single-cell suspension was prepared following previously published methods [33]. Briefly, aortic tissues were digested in 1 mL collagenase cocktail containing 600 μL 1× HBSS, 100 μL collagenase type I (Sigma-Aldrich, C0130, 10× stock: 6750 U/mL), 100 μL collagenase type XI (Sigma Aldrich, C7657, 10× stock: 187.5 U/mL), 100 μL hyaluronidase type I-s (Sigma-Aldrich, H1115000, 10× stock: 900 U/mL), 100 μL DNase I (Sigma-Aldrich, 11284932001, 10× stock: 900 U/mL) for 20 min. The cell suspension was then strained through a 70 μm cell strainer and spun. Pellets from all tissues were subjected to red blood cell lysis and subsequently resuspended in flow cytometry buffer (2% FBS and 0.02% NaN3 in phenol-free DMEM) for further staining. Single-cell suspensions were incubated with JC-1 Dye (T3168, Invitrogen) or Annexin V-FITC and PI (640905, BioLegend, San Diego, CA, USA). After being stained, the cells were passed through a 70 μm filter and sorted on a BD FACSAria Fusion, BD Fortessa, or BD Canto II analyzer. Data were acquired with FACSDiva software (9.0). Cell doublets were excluded by comparison of the side-scatter width to the forward-scatter area.

### 2.7. Transmission Electron Microscopy (TEM)

Samples were prepared by following a modified version of the National Center for Microscopy and Imaging Research (NCMIR) methods for 3D electron microscopy. Initially, samples were fixed in 2.5% glutaraldehyde in 0.1 M sodium cacodylate buffer (pH 7.4) for 2 h at room temperature, followed by post-fixation with 1% osmium tetroxide in the same buffer for 1 h. Samples were then rinsed thoroughly in distilled water. The dehydration process was carried out through a graded series of ethanol (30%, 50%, 70%, 90%, and 100%) for 10 min each and then in 100% acetone twice for 10 min each. For embedding, samples were infiltrated with a mixture of acetone and epoxy resin (1:1) for 1 h, followed by pure epoxy resin overnight. Polymerization was conducted at 60 °C for 48 h. Ultrathin sections (70–90 nm) were cut using a Leica UC7 ultramicrotome and collected on copper grids. The sections were then stained with 2% uranyl acetate in 50% ethanol for 10 min, followed by lead citrate for 5 min. Images were acquired with a JEM 1400 transmission electron microscope (JEOL, Akishima, Tokyo, Japan) operating at an acceleration voltage of 80 kV, equipped with an Ultrascan 1000 CCD camera (Gatan, Inc., Pleasanton, CA, USA). The difference in the length of the d-period for collagen fibrils was measured as described by previously published methods, allowing for detailed morphological analysis and structural characterization of the samples [34].

### 2.8. Aortic Elastin Architecture Preservation and Scanning Electron Microscopy (SEM)

For elastin SEM, mouse thoracic aortas were fixed in 4% paraformaldehyde and then subjected to 90% formic acid treatment for 96 h at 45 °C. Subsequently, they were freeze-dried directly from water, as described in a previously reported study [35]. This preparation method effectively preserved the three-dimensional organization and ultrastructure of the elastin components, allowing for detailed examination via SEM.

For general SEM, samples were prepared by initially fixing in Karnovsky’s fixative kit, which contained 2% paraformaldehyde, 2.5% glutaraldehyde, and 0.1 M sodium phosphate buffer (Fisher Scientific, Pittsburgh, PA, USA), at room temperature for 1 h. After fixation, the samples were rinsed with 0.1 M sodium phosphate buffer to remove excess fixative. The dehydration process involved transferring the samples through a graded series of ethanol solutions (30%, 50%, 70%, 90%, and 100%) for 10 min each. Following dehydration, the samples were dried using a Denton vacuum DCP-1 CO_2_ critical point dryer to preserve their structural integrity by avoiding the surface tension effect that occurs during air drying. To enhance the conductivity of the samples and prevent charging under the electron beam, a thin layer of platinum was sputter-coated on the cross-sections using a Cressington 108 sputter coater (Watford, UK). This step ensured high-quality imaging by reducing artifacts and enhancing the signal-to-noise ratio. The surface morphology of the samples was then examined using a JEOL JSM-6100 SEM (Peabody, MA, USA), operated at an accelerating voltage of 5 kV. This low voltage was chosen to reduce beam damage and charging effects, providing clear and detailed images of the sample surface.

### 2.9. High Throughput RNA Sequencing and Gene Expression Analysis

Myh11-CreERT2 (control, with tamoxifen induction) and ADAR1sm-/- mouse thoracic aortas (6 independent litters in each group) were collected 13 days after the tamoxifen induction for isolated SMCs. Total RNAs were then extracted, and cDNA libraries were constructed from 100 ng of RNA by BGI Genomics. Libraries were sequenced on the NovaSeq 6000 system (Illumina, San Diego, CA, USA). Initial quality control of sequencing data was conducted using FastQC. Subsequently, reads containing Illumina adapters or those of low quality were removed using Trimmomatic. The remaining high-quality reads were then mapped to the mm10 mouse reference genome using the STAR aligner, with all analyses adhering to the standard settings. Gene expression quantification for each library was accomplished using HTSeq. Comparative gene expression analysis across four libraries was conducted using the DESeq2 package (1.36.0) in R (R 4.2.1). Genes showing a fold change greater than 1.5 and an FDR of less than or equal to 5% were classified as differentially expressed genes (DEGs). These DEGs were analyzed using Ingenuity Pathway Analysis to identify significantly enriched pathways, with significance set at an FDR of less than or equal to 5%.

### 2.10. Human Aorta Tissue Specimens

Formaldehyde-fixed human healthy ascending aorta and MFS specimens, embedded in paraffin, were obtained from Mizzou OneHealth Biorepository, School of Medicine, University of Missouri. Human specimens were used under a protocol approved by the Institutional Review Board of the University of Missouri (IRB#2026026). The paraffin-embedded aortic specimens were sectioned and subsequently used for immunostaining as performed in our previous publication [20,29].

### 2.11. TUNEL Analysis

For the analysis of apoptosis in aorta tissue, the Click-iT Plus TUNEL assay kit (C10617, Life Technologies, Carlsbad, CA, USA) was used in accordance with the manufacturer’s instructions. Aorta tissues were fixed in 4% paraformaldehyde in PBS (PFA, AAJ61899AK, ThermoFisher, Waltham, MA, USA) and embedded in Optimal Cutting Temperature compound (OCT, 23-730-571, Fisher, Pittsburgh, PA, USA). Tissue sections (5 µm thick) were prepared for staining. The sections were rehydrated, brought to room temperature, and washed with PBS. After permeabilization, the Click-iT Plus TUNEL reaction was performed as per the protocol provided in the kit. Fluorescence signals indicating apoptotic cells were detected using a Keyence fluorescence microscope. Images with average staining signals were selected as representative pictures and included in the figure panels. The frequency of apoptotic cells in the aorta sections was quantified by determining the percentage of TUNEL-positive cells in five random microscopic fields per specimen.

### 2.12. Statistical Analysis

Each experiment was conducted a minimum of three times, and all data points were independent rather than technical replicates. The D’Agostino and Pearson normality test, with an alpha of 0.05, was used to assess data distribution. For two-group comparisons, the student’s unpaired two-tailed *t*-test was employed for normally distributed data. Statistical analyses were performed using Prism 10.0 (GraphPad Software, San Diego, CA, USA). Data are presented as mean ± SD.

## 3. Results

### 3.1. ADAR1sm-/- Causes Lethality in Mice Due to Extensive Hemorrhages

**Figure 1:**
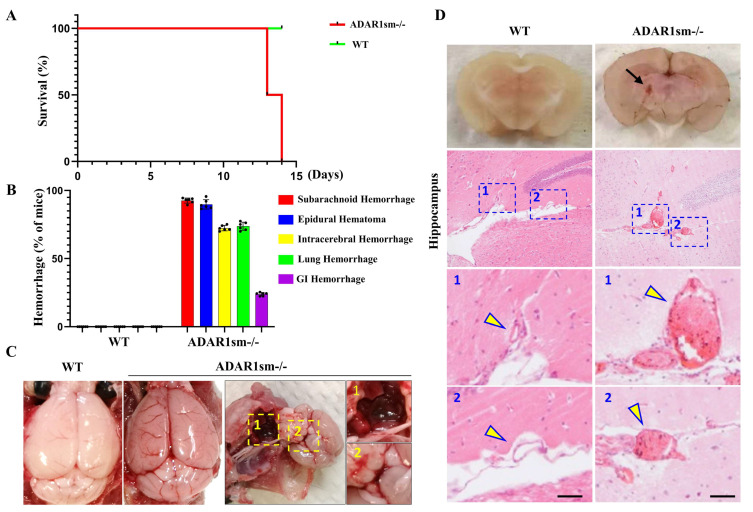
ADAR1 deletion in smooth muscle cells leads to lethality due to vascular defects in mice. Myh11-CreERT2 (WT) and ADAR1sm-/- mice were injected with tamoxifen (1 mg/day, i.p. for 5 days). (**A**) Mouse survival rates post-tamoxifen administration. (**B**) Incidences of hemorrhage in various organs of ADAR1sm-/- mice at 13 days post-injection; n = 6. (**C**) Brain gross images from control and ADAR1sm-/- mice 13 days post-injection. Enlarged panel 1 on the right displays a significant blood clot beneath the brain, indicative of brain herniation. Enlarged panel 2 indicates hemorrhage at the circle of Willis. (**D**) Mouse brain cross-sections show ventricular hemorrhage in ADAR1sm-/- mice. H&E staining reveals small artery dissection causing hemorrhage in ADAR1sm-/- mouse brain hippocampus areas. Scale bar: 50 μm. Lower panels showing enlarged images in the rectangle boxes in the upper panels. Arrows: normal artery in WT mouse brain and diseased artery in brain of ADAR1sm-/- mice.

Following the administration of tamoxifen, we found a surprising and massive death of mice 14 days post-tamoxifen injection (Figure 1A). The dramatically declined survival rate underscores the critical role of ADAR1 in maintaining vascular health and organismal viability. A systematic evaluation revealed widespread hemorrhages in various organs (Figure 1B). Further examination of the brains of these mice uncovered severe hemorrhagic events, particularly beneath the brain surface and at the circle of Willis, indicating rupture of brain vasculatures (Figure 1C). These observations were further supported by brain cross-sections, which showed ventricular hemorrhage and small artery dissection within the brain tissue of ADAR1 sm-/- mice (Figure 1D). Hematoxylin and eosin (H&E) staining of these sections highlighted the structural deteriorations that may have contributed to these vascular anomalies (Figure 1D). These data indicate that ADAR1 plays a vital role in maintaining the structural integrity of blood vessels.

### 3.2. ADAR1sm-/- Impairs Elastin Fiber Integrity

**Figure 2:**
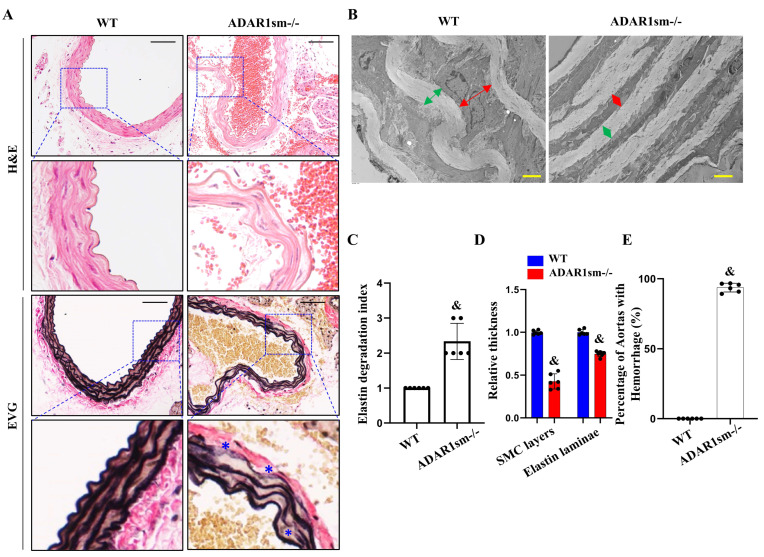
ADAR1 deletion in smooth muscle cells damages the integrity of elastin fibers. Myh11-CreERT2 (WT) and ADAR1sm-/- mice were injected with tamoxifen (1 mg/day, i.p. for 5 days). (**A**) H&E and EVG staining of thoracic aorta sections of WT and ADAR1sm-/- mice 13 days post-tamoxifen injection. * Breaks in elastin fibers. Scale bar: 50 μm. (**B**) Transmission electron microscopy (TEM) images of WT and ADAR1sm-/- mouse aorta segments 13 days post-tamoxifen injection. Green arrows indicate the thickness of elastin lamina, and red arrows the thickness of SMC layer. Scale Bar: 4 μm. (**C**) Quantification of the elastin degradation index in WT and ADAR1sm-/- mouse aortas. ^&^ *p* < 0.001 vs. WT, n = 6. (**D**) Quantification of the SMC layer and elastin laminae thicknesses in WT and ADAR1sm-/- mouse aortas. ^&^ *p* < 0.01 vs. WT, n = 6. (**E**) Quantification of hemorrhage events in aortas of WT and ADAR1sm-/- mice. ^&^ *p* < 0.001 vs. WT, n = 6.

To determine how ADAR1sm-/- causes vascular defects, we analyzed the potential structural damages in large arteries. Histological analyses of thoracic aortas by hematoxylin and eosin (H&E) and elastica van Gieson (EVG) staining revealed notable elastin fiber degradation or disruptions in ADAR1sm-/- mouse aortas compared with wild-type (WT) controls (Figure 2A). Transmission electron microscopy (TEM) revealed distorted and thin elastin laminae as well as narrowed SMC layers in ADAR1sm-/- mouse aortas (Figure 2B). The elastin degradation index analyses indicated enhanced elastin breakdown in ADAR1sm-/- mouse aortas compared with controls (Figure 2C). Moreover, the thicknesses of SMC and elastin layers were significantly reduced in ADAR1sm-/- mouse aortas (Figure 2D). This morphological evidence indicates a compromised arterial structure with media layer degeneration in ADAR1sm-/- aortas. Indeed, a significantly higher percentage of hemorrhage events were observed in ADAR1sm-/- aortas compared with WT (Figure 2E), further underscoring the functional consequences of the damaged vascular integrity.

### 3.3. ADAR1sm-/- Impairs the SMC and Elastic Laminae Interaction

**Figure 3:**
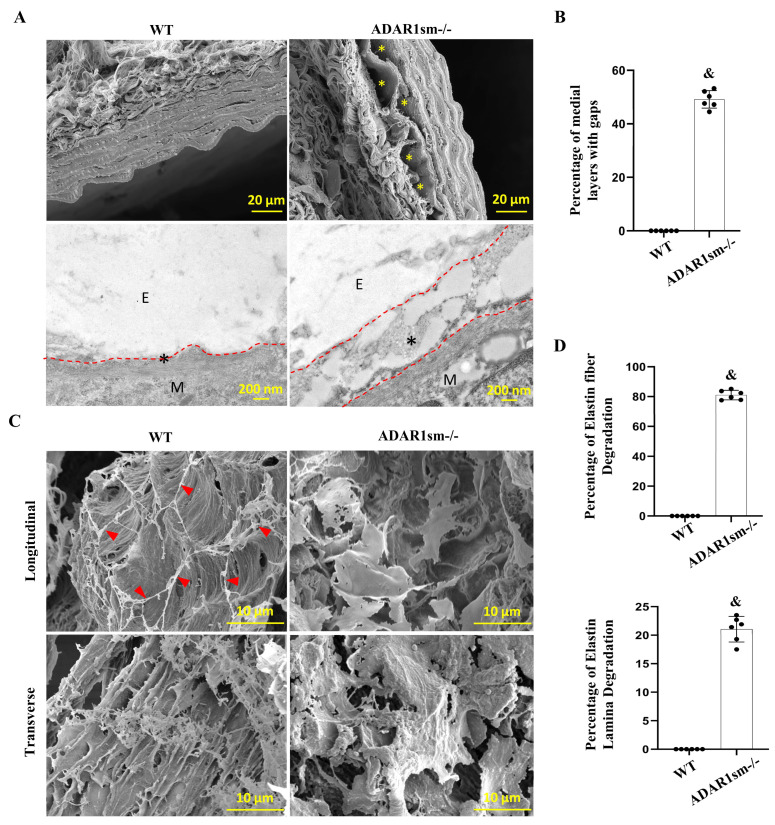
ADAR1 deletion causes detachment of smooth muscle layers from elastic fibers. Myh11-CreERT2 (WT) and ADAR1sm-/- mice were injected with tamoxifen (1 mg/day, i.p. for 5 days). Thirteen days later, descending thoracic aortas were analyzed by electron microscopy. (**A**) Scanning electron microscopy images of WT and ADAR1sm-/- mouse aorta media layers, * gaps between elastin lamina and SMC layers (upper panels). The microstructure of the extracellular matrix (ECM) in the descending aorta was observed by transmission electron microscopy. ‘E’ indicates elastin lamina; ‘M’ indicates SMCs; asterisks (*) highlight the gaps between elastin lamina and SMCs (lower panels). (**B**) Quantification of the percentage of medial layers with gaps between elastin lamina and SMCs. ^&^ *p* < 0.001 vs. WT, n = 6. (**C**) Scanning electron microscopy (SEM) of the descending aorta segments after formic acid digestion and freeze-drying, which preserved only the elastin. Red arrows indicate elastin fibers in WT tissues, which is absent in ADAR1SM-/- aortas, shown in longitudinal view. The transverse view shows the degradation of elastin lamina in ADAR1sm-/- aortas. (**D**) Quantification of the degradation of elastin fibers connecting different layers of elastin laminae. ^&^ *p* < 0.001 vs. WT, n = 6.

**Figure 4:**
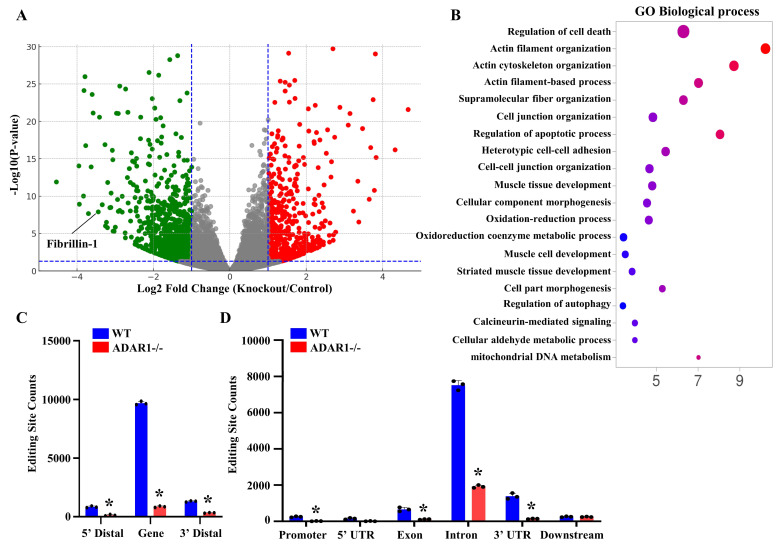
RNA-seq analyses reveal critical signaling pathways and genes altered by ADAR1sm-/- in aortic SMCs. Myh11-CreERT2 (WT) and ADAR1sm-/- mice were injected with tamoxifen (1 mg/day, i.p. for 5 days). Thirteen days later, SMCs from thoracic aortas were isolated, total RNAs were extracted, and bulk RNA sequencing was conducted. (**A**) Volcano plot shows that numerous genes were up- (red dots) or downregulated (green dots) by ADAR1sm-/- (adjusted *p*-value < 0.01, log2 fold change > 1). Six independent samples from each group were analyzed. (**B**) KEGG pathway enrichment analyses of differentially expressed mRNAs in ADAR1SM-/- mice compared with WT mice, based on transcriptomic data from 6 mouse aortas per group. (**C**) Editing site distribution in aortic SMCs based on sequencing data. Most editing sites are within gene regions, particularly the introns and 3′ untranslated regions, with ADAR1sm-/- significantly reducing editing site counts in these regions. * *p* < 0.01 vs. WT in each group, n = 3. (**D**) Approximately half of the editing sites are in the repetitive regions, primarily within the short interspersed nuclear elements, long terminal repeats, and long interspersed nuclear elements. ADAR1sm-/- substantially abolishes the editing sites in all these regions. * *p* < 0.01 vs. WT in each group, n = 3.

Scanning electron microscopy (SEM) images provided a visualization of the void spaces between elastin laminae (Figure 3A, upper panels). A closer observation with high-power TEM showed a clear view of the microstructure within the ECM, and notable gaps were observed between the elastin lamina (indicated by ‘E’) and SMC layers (indicated by ‘M’) in ADAR1sm-/- mouse aortas (Figure 3A, lower panels). These gaps (highlighted with asterisks *) indicate a loss of attachment between these critical structural components and compromised structural integrity of the vessel wall, which could predispose to vascular dysfunction and pathology. Quantitative analyses of the extent of these structural disruptions revealed that the percentage of medial layers with gaps between elastin lamina and SMCs was dramatically increased in ADAR1sm-/- aortas compared with WT controls, underscoring the detrimental effect of ADAR1sm-/- on ECM cohesion (Figure 3B). Further structural insights were gained through SEM following the depletion of SMCs while selectively preserving elastin structure with formic acid digestion and freeze-drying of the descending aorta. As shown in Figure 3C, ADAR1sm-/- caused pronounced degradation of elastin fibers that run through SMC layers and connect different elastin laminae layers surrounding SMCs (Figure 3C, red arrowheads, longitudinal views). The transverse views further illustrated the degradation and distortion of the elastin lamina in ADAR1sm-/- aortas (Figure 3C). Quantitative analyses revealed a massive elastin fiber and elastin lamina degradation in ADAR1sm-/- aortas (Figure 4D). These findings collectively indicate that ADAR1 is essential for maintaining the structural cohesion between SMCs and elastin laminae in vascular ECM.

### 3.4. ADAR1sm-/- Alters the Expression and RNA Editing Profiles of Genes Related to Vascular Homeostasis

To determine the molecular mechanisms underlying ADAR1sm-/- arterial structure defects, we performed transcriptomic analyses for SMCs isolated from WT and ADAR1sm-/- mouse aortas via RNA sequencing. A volcano plot illustrated numerous genes that were up- or downregulated by ADAR1sm-/-, including a notable downregulation of Fibrillin-1, one of the important structural components in artery walls. Further Kyoto Encyclopedia of Genes and Genomes (KEGG) pathway enrichment analyses highlighted several biological pathways significantly impacted by ADAR1sm-/- (Figure 3B). These pathways are closely related to vascular functions and artery structural organization. RNA-seq analyses also revealed that ADAR1sm-/- profoundly diminished RNA editing, the key function of ADAR1. Most editing sites appeared to be within gene regions, especially in introns and 3′ untranslated regions (UTRs). The absence of ADAR1sm-/- SMCs exhibited dramatic reductions in editing activities in these sites (Figure 3C). Moreover, a significant proportion of editing occurred in repetitive regions of the genome, such as short interspersed nuclear elements (SINEs), long terminal repeats (LTRs), and long interspersed nuclear elements (LINEs). ADAR1sm-/- almost completely abolished RNA editing in these regions (Figure 3D), highlighting the importance of ADAR1 in maintaining genome stability and proper gene expression regulation in SMCs. These findings collectively indicate that ADAR1 is crucial not only for the structural integrity of artery walls but also for SMC functions and vascular health.

### 3.5. ADAR1sm-/- Leads to SMC Apoptosis through the Mitochondrial Pathway

**Figure 5:**
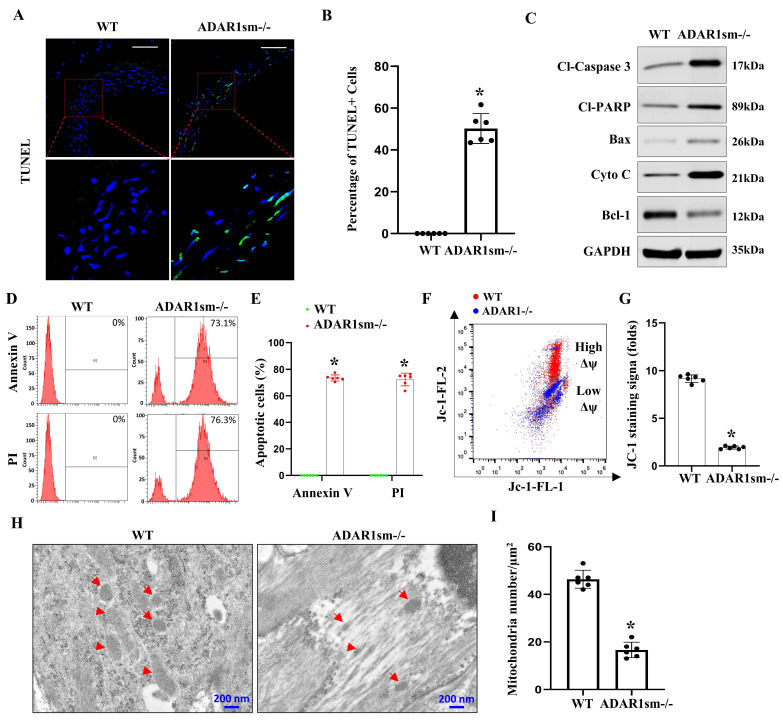
ADAR1sm-/- causes SMC apoptosis in aortas via mitochondrial pathway. Myh11-CreERT2 (WT) and ADAR1sm-/- mice were injected with tamoxifen (1 mg/day, i.p. for 5 days). Thirteen days post-tamoxifen injection, descending thoracic aortas were isolated and processed. (**A**) TUNEL staining of aorta sections from WT and ADAR1sm-/- mice. Scale bar: 40 μm. (**B**) Quantification of TUNEL-positive cells. * *p* < 0.001 vs WT, n = 6. (**C**) Cleaved Caspase-3 (Cl-caspase3), cleaved PARP (Cl-PARP), BCL-1, Bax, cytochrome C, and GAPDH levels in WT and ADAR1sm-/- mouse aortic media (adventitia removed) analyzed by Western blot. (**D**) Flow cytometry assessment of PI and Annexin V staining of the single-cell suspensions from aorta media layers. (**E**) Quantification of PI- and Annexin V-positive cells (percentages of total cells) for each group. * *p* < 0.001 vs WT, n = 6. (**F**) Flow cytometry assessment of JC-1 staining of single-cell suspensions from aorta media layers. Red dots indicate high mitochondrial membrane potential in WT SMCs, and blue dots show low membrane potential in ADAR1sm-/- SMCs. (**G**) Quantification of JC-1 staining, shown as fold changes. * *p* < 0.001 vs WT, n = 6. (**H**) TEM images of aorta media layers of WT and ADAR1sm-/- mice 14 days post-tamoxifen injection. Red arrows indicate mitochondria. (**I**) Quantification of mitochondria numbers per square micron, * *p* < 0.001 vs WT, n = 6.

Since ADAR1sm-/- caused medial layer degeneration, we sought to determine if ADAR1 is important to SMC survival in the aorta. TUNEL staining of the descending aorta highlighted a significant increase in SMC apoptosis in ADAR1sm-/- mouse aortas compared with WT controls (Figure 5A). Quantitative analyses showed that nearly 60% of SMCs in ADAR1-/- aortas were TUNEL-positive (Figure 5B), indicating a critical role of ADAR1 in SMC survival.

Western blot analyses showed increased protein levels of cleaved Caspase-3, cleaved PARP, Bax, and Cytochrome C but decreased BCL-1 expression in ADAR1sm-/- aortas (Figure 5C), suggesting the activation of mitochondrial apoptotic machinery by ADAR1sm-/- in SMCs. Flow cytometry analyses using PI and Annexin V staining of single-cell suspensions from the aorta media layers 13 days post-tamoxifen injection revealed dramatically higher apoptosis rates in ADAR1sm-/- aortas than WT aortas (Figure 5D,E), consistent with the results observed with TUNEL staining. Further investigations using JC-1 staining to assess mitochondrial membrane potential demonstrated a decrease in membrane potential in ADAR1sm-/- SMCs, as indicated by a shift from red to blue fluorescence, which signals mitochondrial depolarization and an increase in apoptotic cell populations (Figure 5F,G). Moreover, TEM imaging of the aortas revealed structural changes in mitochondria, with increased mitochondria damage in ADAR1sm-/- SMCs (Figure 5H,I), further confirming the involvement of mitochondrial dysfunction in the observed SMC apoptosis. Collectively, these results demonstrated that ADAR1sm-/- leads to SMC apoptosis through the mitochondrial pathway, highlighting ADAR1 as a protective factor against mitochondrial dysfunction and apoptosis in vascular cells.

### 3.6. ADAR1sm-/- Disrupts the Fibrillin-1 and Elastin Interaction in the Aorta ECM

**Figure 6:**
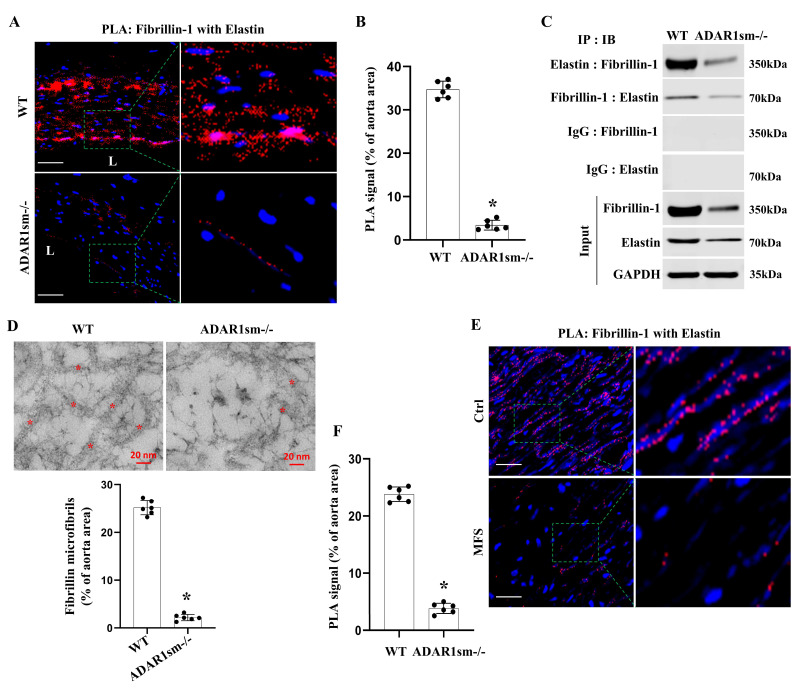
ADAR1 deletion in smooth muscle disrupts the fibrillin–1-elastin interaction in aorta media layers. Myh11-CreERT2 (WT) and ADAR1sm-/- mice were injected with tamoxifen (1 mg/day, i.p. for 5 days). Thirteen days post-tamoxifen injection, descending thoracic aortas were isolated and processed. (**A**,**B**) In situ proximity ligation assays (PLAs) were conducted to assess fibrillin-1–elastin interactions in WT and ADAR1sm-/- mouse aortas. DAPI stains the nuclei. Scale bar: 20 μm. (**B**) Quantification of PLA signals (percentages of aorta areas). * *p* < 0.001 vs WT, n = 6. (**C**) Coimmunoprecipitation assays detecting fibrillin-1–elastin interactions in mouse aorta media layers. Normal IgG (control), fibrillin-1, or elastin antibodies were used for immunoprecipitation (IP), followed by immunoblotting (IB) with antibodies against fibrillin-1 and elastin. (**D**) High-magnification TEM images of the aorta media layers of WT and ADAR1sm-/- mice. Asterisks (*) indicate fibrillin microfibrils, which were quantified as percentages of aorta areas. * *p* < 0.001 vs WT, n = 6. (**E**) In situ proximity ligation assays (PLAs) confirmed fibrillin-1–elastin interactions in healthy human (Ctrl) and Marfan Syndrome (MFS) patients’ aorta media layers. DAPI stains the nuclei. Scale bar: 20 μm. (**F**) Quantification of PLA signal (percentages of aorta areas) shown in (**E**). * *p* < 0.001 vs Ctrl, n = 6.

To further study how ADAR1sm-/- caused alterations in the ECM of the aortas, we detected the physical interactions between fibrillin-1 and elastin, the critical proteins for maintaining vascular integrity, by using the in situ proximity ligation (PLA) assay. Intensive fibrillin-1–elastin interactions were observed in WT aorta media layers (Figure 6A,B). However, the interactions largely disappeared or decreased in ADAR1sm-/- aortas, as evidenced by the sparse PLA signal (Figure 6A,B). Coimmunoprecipitation assays further confirmed the significantly decreased binding of fibrillin-1 with elastin in ADAR1sm-/- aorta protein extracts (Figure 6C). Their reduced interaction points to potential destabilization of the aortic ECM. Indeed, TEM imaging revealed the disrupted ECM microstructure, especially the disruption and reduction of fibrillin microfibrils in ADAR1sm-/- aortas (Figure 6D). These data suggest that ADAR1 plays a crucial role in stabilizing artery ECM protein complexes.

Since fibrillin-1 defect is related to Marfan Syndrome, a human genetic disorder that severely impacts connective tissues, we detected if the fibrillin-1 interaction with elastin is also altered in the aortas of Marfan patients. As shown in Figure 6E,F, intensive PLA signals indicative of the fibrillin-1–elastin interaction were observed in the aortas of healthy individuals. However, the interactions were severely diminished in the aortas of Marfan patients Figure 6E,F. These data highlight the importance of fibrillin-1 and elastin cohesion in maintaining aortic structural integrity in the human aorta, and the disrupted fibrillin-1–elastin interactions may be one of the potential molecular mechanisms contributing to the aortic abnormalities in Marfan Syndrome.

## 4. Discussion

Artery structural integrity is critically important for vascular homeostasis. SMCs play crucial roles in maintaining artery structural integrity. The roles of SMCs in artery wall homeostasis are multi-dimensional. On the one hand, SMCs are important building blocks supporting blood vessel wall structure. Preservation of healthy and functional SMCs is essential for the physiological functions of blood vessels. On the other hand, SMCs play fundamental roles in maintaining the homeostasis of ECM and ECM protein interactions. Our data show that the absence of ADAR1 causes SMC apoptosis, SMC detachment from elastin laminae, and dissociation of elastin from fibrillin, demonstrating that ADAR1 is not only important for SMC survival, which causes medial layer degeneration, but also essential for SMC function in maintaining its interaction with ECM proteins and the interactions among ECM proteins. Overall, our study establishes ADAR1 as an essential regulator of vascular homeostasis.

ADAR1 appears to affect SMC survival through both molecular and cellular mechanisms. Since RNA editing is critically important for the splicing of pre-mRNAs to mature mRNAs of numerous genes, the reduction in RNA editing in ADAR1-deficient SMCs is likely to cause the impaired expression of genes promoting SMC survival. Indeed, RNA sequencing analyses reveal that ADAR1 loss results in significant reductions in RNA editing in genes crucial for maintaining genome stability and proper gene regulation. In addition, ADAR1 loss promotes Bax while reducing Bcl-1 expression, causing cytochrome C release and suggesting that ADAR1 protects SMC from cell death by inhibiting the mitochondrial apoptosis pathway. Moreover, the detachment of SMCs from elastin laminae is likely to cause stress to SMCs, which may exacerbate SMC apoptosis.

ADAR1 deficiency in SMCs disrupts ECM integrity likely by downregulating both elastin and fibrillin-1. Although it is unclear whether ADAR1 regulates elastin and fibrillin-1 through RNA editing or its non-editing function, the reduced expression of these two proteins not only reduces their interaction but also may cause the dissociation of SMCs from elastin laminae as well as the disruption of fibrillin microfibrils. This collectively damages ECM stability, significantly increases the vulnerability of the blood vessel wall, causes rupture of the artery wall, especially in small arteries, and eventually leads to widespread hemorrhage and animal death. In the large artery, the disruption of the ECM structure and the apoptosis of SMCs causes progressive degeneration of both elastin laminae and medial SMCs, which would continuously weaken the artery wall and also lead to rupture. Our study is relevant to human aorta pathology because the aorta of patients with Marfan Syndrome appears to exhibit similar disruption of fibrillin-1–elastin interactions. Whether or not this decreased interaction in the human aorta is due to ADAR1 deficiency requires additional study in the future.

It is interesting that ADAR heterozygous deficiency in SMCs is protective [29], while ADAR1 homozygous deficiency in SMCs has detrimental effects. Our prior and current studies suggest that a proper level of ADAR1 is critically important for human or animal health. Excessive ADAR1, as observed in injured arteries or during aneurysm development, exacerbates pathological artery wall remodeling. The absence of ADAR1 in SMCs causes even more severe consequences, i.e., degeneration of the vascular system and lethality. These are likely to be attributed, at least partially, to its essential RNA editing function affecting a very large number of mammalian genes and its numerous non-editing functions. Therefore, selectively targeting a specific function of ADAR1, especially its non-editing function, in a particular cell population could be a feasible strategy to combat diseases driven by the excessive expression of the ADAR1 protein.

Taken together, our study underscores the critical role of ADAR1 in maintaining vascular structural integrity and highlights its profound implications for vascular health and stability. The absence of ADAR1 in SMC results in severe disruptions to vascular architecture and increased susceptibility to hemorrhage because of SMC death and disruption of ECM homeostasis, especially fibrillin-1 and elastin expression and their interactions. This study lays the groundwork for a deeper understanding of the molecular interactions within vascular walls that are vital for maintaining the mechanical properties required for normal vascular function and organismal viability.

## Data Availability

The original contributions presented in this study are included in this article. Further inquiries can be directed to the corresponding author.

## Supplementary Materials

The following supporting information can be downloaded at: https://www.mdpi.com/article/10.3390/cells13151257/s1, Original Images for Blots.
