# Supplementary figures and images for: ADAR1 Is Essential for Smooth Muscle Homeostasis and Vascular Integrity
Source: Cells. 2024 Jul 26;13(15):1257. doi: 10.3390/cells13151257 (PMC11311430; doi:10.3390/cells13151257)

Original Images for Blots

Figure 5C

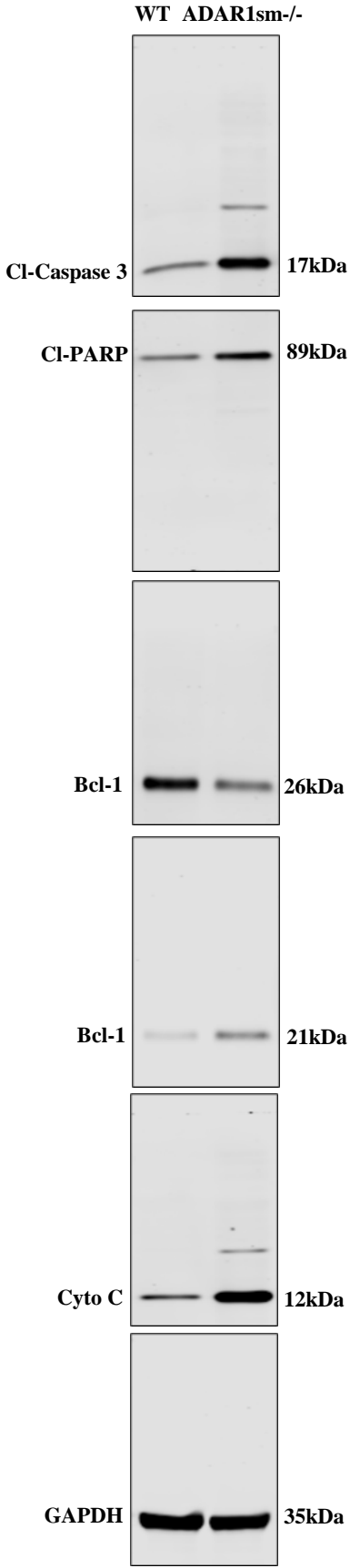

**Figure 6C**

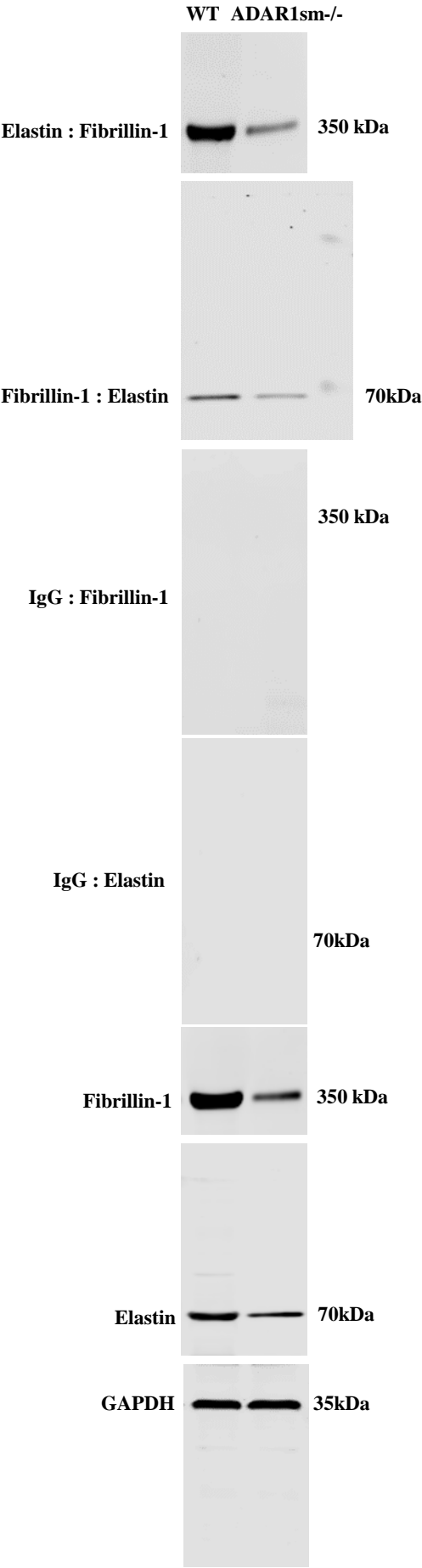

Supplement: Supplementary file 1 [file cells-13-01257-s001.zip › cells-3069817-supplementary.pdf]
